# Malaria and the mobile and migrant population in Cambodia: a population movement framework to inform strategies for malaria control and elimination

**DOI:** 10.1186/s12936-015-0773-5

**Published:** 2015-06-20

**Authors:** Philippe Guyant, Sara E Canavati, Nguon Chea, Po Ly, Maxine Anne Whittaker, Arantxa Roca-Feltrer, Shunmay Yeung

**Affiliations:** Department of Global Health and Development, Malaria Centre, London School of Hygiene and Tropical Medicine, London, UK; Malaria Consortium, Phnom Penh, Cambodia; National Center for Parasitology, Entomology and Malaria Control, Phnom Penh, Cambodia; Partners for Development, Phnom Penh, Cambodia; Department of Clinical Tropical Medicine, Faculty of Tropical Medicine, Mahidol University, Bangkok, Thailand; School of Public Health, University of Queensland, Brisbane, Australia

**Keywords:** Framework, Migrants, Mobile populations, Cambodia, Artemisinin resistance, Malaria elimination, Strategy, Index

## Abstract

**Background:**

The relationships between human population movement (HPM) and health are a concern at global level. In the case of malaria, those links are crucial in relation to the spread of drug resistant parasites and to the elimination of malaria in the Greater Mekong sub-Region (GMS) and beyond. The mobile and migrant populations (MMP) who are involved in forest related activities are both at high risk of being infected with malaria and at risk of receiving late and sub-standard treatment due to poor access to health services. In Cambodia, in 2012, the National Malaria Control Programme (NMCP) identified, as a key objective, the development of a specific strategy for MMPs in order to address these challenges. A population movement framework (PMF) for malaria was developed and operationalized in order to contribute to this strategy.

**Methods:**

A review of the published and unpublished literature was conducted. Based on a synthesis of the results, information was presented and discussed with experienced researchers and programme managers in the Cambodian NMCP and led to the development and refinement of a PMF for malaria. The framework was “tested” for face and content validity with national experts through a workshop approach.

**Results:**

In the literature, HPM has been described using various spatial and temporal dimensions both in the context of the spread of anti-malarial drug resistance, and in the context of malaria elimination and previous classifications have categorized MMPs in Cambodia and the GMS through using a number of different criteria. Building on these previous models, the PMF was developed and then refined and populated with in-depth information relevant to Cambodia collected from social science research and field experiences in Cambodia. The framework comprises of the PMF itself, MMP activity profiles and a Malaria Risk Index which is a summation of three related indices: a vulnerability index, an exposure index and an access index which allow a qualitative ranking of malaria risk in the MMP population. Application of currently available data to the framework illustrates that the highest risk population are those highly mobile populations engaged in forest work.

**Conclusion:**

This paper describes the process of defining MMPs in Cambodia, identifying the different activities and related risks to appropriately target and tailor interventions to the highest risk groups. The framework has been used to develop more targeted behaviour change and outreach interventions for MMPs in Cambodia and its utility and effectiveness will be evaluated as part of those interventions.

## Background

### Human population movement (HPM) and health

Migration and health have become a major concern in the last few years in the context of globalization and has drawn attention from policy makers both from governments and from international institutions [1, 2]. In 2010, at global level, it was estimated that migrants represented almost one billion people, consisting of 214 million international migrants (40% moving between neighbouring countries) and 740 million internal migrants [1]. The most general definition of “migrants” refers to individuals who changed their usual place of residence for more than 1 year. This can be refined and adjusted depending on the lens used to look at population movement [2]. In addition to people who may, on a more longer term basis, move primary place of residence (migrate), there are also people who are mobile for short periods of time, for work, cultural, social or tourism reasons. In this paper, the term HPM is used when referring to the processes involved in population movement and mobile and migrant population (MMP) when referring to people (individuals) in movement, although this is not a homogeneous or fixed group [3]. HPM in relation to health outcomes and potential health threats (emerging or re-emerging diseases) is a global concern, fuelled by globalization and demographic and socio-economic disparities [1, 2, 4, 5]. From a global health perspective, population movement, has been and continues to be considered one of the main drivers of major infectious disease transmission, as MMP are exposed to higher risks of infectious diseases or risks of not receiving adequate treatment compared to non-migrant population [4]. This is illustrated historically by plague and cholera and related quarantine measures and more recently by the severe acute respiratory syndrome (SARS) in 2003, the influenza H1N1 pandemic in 2009 and the Ebola outbreak in 2014 [4]. Three main factors need to be considered when looking at migration and health: (1) disparity of health environments (2) movement of population between regions of different prevalence of health indicators (3) vulnerability of migrants population during the various phases of migration [6]. The complexity of the migration processes, the lack of common terminology, the importance of health determinants (biological, behavioural, environmental and socio-economic) have led to the development of population-based frameworks to inform policy-makers and strategies relating to the migrant population, at various spatial and temporal scales [2, 4, 7]. Population movements can be categorized according to spatial and temporal characteristics: spatially, migration can occur within a country (rural/urban, rural/rural and urban/urban) or between countries (contiguous and non-contiguous international movement) [8]; while temporally, distinctions are made between migration (permanent/very long term change of residence) and circulation (shorter-term and cyclical movements, no change of residence) [9]. As defined by Gushulak [6]: “A population health-based approach considers the relationship between migration and health as a progressive, interactive process influenced by temporal and local variables”.

### HPM and malaria in the Greater Mekong sub-Region (GMS)

Population movement has historically posed a huge challenge to the control and elimination of malaria. In 1957, at the time of the Global Malaria Eradication Programme, the WHO Malaria Expert Committee stated that “mass movements within or through a malarious country in the malaria season are likely to cause an exacerbation of the disease to the extent of often precipitating a severe epidemic”. [10]. More recently, following the renewal of the malaria elimination paradigm [11, 12], the critical need to address population movement to achieve and sustain malaria elimination has been recognized in view of the central role it plays in the reintroduction of imported cases into malaria-free areas and in the spread of drug resistant parasites to new areas [8, 13–17]. In relation to malaria, Prothero and others have described the importance of the distinction between migration and circulation, and the need to apply various temporal and spatial dimensions to distinguish different categories of human mobility depending on seasonality of agricultural or forest related activities [9, 18]. These concepts have been applied to describe mobility patterns in northern Thailand [9, 19]. The recent identification of artemisinin drug resistance on the Thai–Cambodia border along with the renewed calls for the elimination of malaria have once again, brought to the fore the relevance of HPM to National Malaria Control Programmes (NMCPs) and stakeholders in the region [13, 20, 21]. These have led to further studies based on the typology of HPM developed by Prothero and aimed at inform global, regional and national strategies both in the context of the spread of anti-malarial drug resistance, and malaria elimination [7, 8, 16, 22–26].

In the World Health Organization’s Global Plan for Artemisinin Resistance Containment (GPARC), operational research into MMPs is highlighted as a vital part of containing and preventing resistance and this has been further emphasized in the Emergency Response to Artemisinin Resistance (ERAR) framework [14, 15]. According to the strategy document, building scalable models to reach MMPs should be the highest priority for research. In the GMS, individuals moving from areas of high to low transmission hinder control and elimination of malaria by importing infections and acting as sources of local transmission, while facilitating the spread of drug resistance parasites [27–30]. High frequency of cross-border movement has been documented between Cambodia and its neighbours: Thailand, Laos and Vietnam [27, 29]; and the frequency of border-crossing among Cambodian people has previously been associated with malaria infection [31].

### Drivers of HPM in Cambodia

The concept of “push and pull factors” has been used to better understand the factors affecting population movement [2, 6, 29, 30, 32, 33]. In Cambodia, as elsewhere in Asia [34], poverty is closely related to migration, with most internal migration being due to economic reasons [35–37]. Initially pushed to migrate due, for instance to landlessness (sometimes related to catastrophic health expenditures) or lack of economic opportunities at the place of origin, MMPs at their destination still lack land ownership, proper housing and basic assets, and have access only to non-permanent or short-term jobs, “3D jobs (Dirty, Dangerous and Disliked)” which allow them only to maintain the status quo rather than improving their standard of living [36]. Land use and land resources are therefore the main drivers of population movement from the densely populated central areas to the less densely populated forested and border areas, rich in natural resources [35–37]. Mobile populations come to the new place, attracted by land development, with a variety of purposes which include farming work, mining, investment, trade, visiting relatives, and eventually the prospect of finding a new settlement [35]. Poverty affects families in both the place of origin as a push factor and at the place of destination where migrants and mobile populations can get caught further in a poverty cycle, especially when as non-immune individuals they are exposed to malaria.

### MMP, vulnerability and malaria

Vulnerability is a complex concept and has been used in different settings (disaster management, climate change, poverty, HIV/AIDS). Bates et al. described vulnerability in terms relevant to malaria and MMPs as: “Vulnerability encompasses the factors that lead to variation in the impact of disease between different communities and individuals”. Those factors have been identified at various levels: individual level (biological and disease related factors); household and community level (social and economic factors); meso/macro level (environmental and institutional factors) [38].

At a macro level, the malaria ecosystem in Cambodia, as in most of South-East Asia, is mainly related to the forests, and has been described as “forest malaria” [39]. This is because the main malaria vectors in Cambodia are forest vectors: *Anopheles dirus* (usually found in thick forest or forest fringe) and *Anopheles minimus* (present in edges of flowing waters such as foothill streams, and springs). The highly anthropophilic and exophagic characteristics of *An. dirus* combined with early biting behaviour makes it a highly efficient malaria vector and raises the issue of outdoor and residual transmission and the importance of the type of housing. Although the size of forested areas has drastically reduced over the past few years, forest-related activities are still important sources of income for a significant proportion of Cambodians. Therefore at an individual level, individuals living close to the forest and forest goers, including those staying overnight in forest huts, have a higher risk of being parasitaemic than people further away from forest or village residents [40–44]. Working primarily in the forest or residing in the forest have also been identified as important risk factors for malaria infection in Vietnam [45–48] and Thailand [49–52]. Housing types (types of wall, roof) and constructions conditions have been shown to be associated with mosquito entry, and individuals living in poorly constructed houses, bamboo or mud houses have been found to be at higher risk of malaria than those living in wooden, concrete or cement houses [46, 53–61]. Housing conditions in forest settings are often basic; sometimes there are no houses so temporary visitors simply sleep in a hammock between two trees.

At the household and community level, malaria has been found to affect the poorest of the poor with 58% of malaria deaths occurring among the 20% poorest of the world population [62], although a review published in 2005 found mixed evidence on the link between poverty and malaria incidence at individual and households levels [63]. A more recent systematic review found that the odds of malaria infection among the poorest children was higher than among the least poor [64]. More specifically in South-East Asia, a higher risk of infection has been shown among the poorest and among forest goers or migrants seeking work in the forest in Cambodia [41, 42], Thailand [65, 66] and Vietnam [47, 48].

MMPs are biologically more vulnerable to malaria because they often come from non-forested areas where they are not exposed to malaria, to forested areas where they are. Compared to the local population who will have developed a relative immunity to malaria through repeated exposure, non-immune travellers and migrants, bitten by an infected mosquito, have a higher risk of becoming parasitaemic, having a high parasitaemia, clinical malaria, severity and death [9, 32, 38, 67–71]. This increase in risk has been described in the context of forest malaria, in Thailand [19, 50, 72], Cambodia [41], India [73], and Brazil [74]. Studies conducted in Indonesia among migrants from Java to Irian Jaya demonstrated that in such a situation, non-immune migrants would develop protective immunity towards malaria within 12–24 months after moving to the new area [75, 76].

Education and knowledge are key factors in influencing malaria prevention and treatment-seeking behaviour although the relationship between knowledge and behaviour is complex and the result of the interaction of social, cultural and economic factors [35, 38, 69]. Mobile and migrant population are less likely to be aware of existing health services than local long term residents [29, 34, 68, 77] and if they arrive from non-malaria areas are less likely to have heard health education messages for malaria than local population living in malaria transmission areas [35, 78]. In the Mekong Subregion, poor knowledge of malaria transmission and prevention has been shown to be a risk factor of malaria infection [48, 50, 65, 66].

The effectiveness of insecticide-treated nets (ITN) in reducing malaria morbidity and mortality is clearly recognized [79] and the universal coverage of this intervention is now one of the two main pillars of the malaria control strategy globally [12]. However ownership and actual use of different types of prevention measures will affect exposure to mosquito bites [61, 80–82]. There is evidence that ITNs are effective in protecting migrants and/or forest goers, and that conversely the lack of ownership or use of ITN or insecticide-treated hammock nets (ITHN) is a risk factor of infection as shown in Thailand [51, 52, 66, 83, 84] and Vietnam [45–47]. Furthermore, the use of ITHN by forest goers has been shown to reduce incidence and prevalence among forest goers in Vietnam [85] and to reduce mosquito bites in Cambodia [86]. However, in the Cambodia Malaria Survey 2010, the use of nets by forest goers, travellers or visitors was slightly lower than the general population and the proportion of forest goers, living more than 2 kms from the forest, using a long lasting insecticide net (LLIN) when they stayed overnight in the forest was low at 13.6% [42].

Since the Alma Ata declaration [87] the importance of the access in determining use of health services use has been well recognized. The concept of “access” is often described as consisting of the following dimensions: availability, accessibility, accommodation, affordability and acceptability [88–90]. Distance, or more importantly, travel time from population settlements to health facilities or health providers are an important component of access to health care and have been described or modelled in various settings [91–94]. This issue is particularly important for mobile and migrants population working in remote forested areas, in Cambodia and in the GMS and constitute one of the major barriers to reaching these population and for them to access diagnosis and treatment services [28, 29, 34, 78, 95–97]. The high mobility of the MMP is known as one of the main limitations for malaria control and elimination in the GMS and the mobility of the work location has been identified as an important determinant of access and outreach [28, 95, 98].

Many of these factors contribute to the high incidence of malaria amongst MMPs in Cambodia when compared with the more “static or less mobile” population of similar socio-economic and demographic profile typically captured in standard household surveys: the results of a national malaria survey conducted in Cambodia in 2010 showed that the prevalence of malaria among mobile populations (including travellers, visitors, and forest goers) is generally higher than the resident population and that the odds of having a positive blood slide increase three-fold for forest goers compare to non-forest goers [42].

To address this challenge and to support the goals of the National Malaria Elimination Strategy 2025, the Cambodian NMCP proposed the development of an MMP strategy aimed at adapting and better targeting interventions to these hard-to reach populations [99]. In order to contribute to this process and as a first step towards further planning and research, a population movement framework (PMF) was developed in the context of malaria in Cambodia. This was needed to differentiate between different types of MMPs, based on key characteristics that would help to determine the most effective strategies to target and reach these populations with the most appropriate interventions. The process involved characterising and defining the MMPs in Cambodia, identifying the different activities and risks as well as the types of intervention strategies needed to appropriately target this high malaria risk group.

This paper describes the process and the resulting framework, starting with a formulation of the key research questions based upon the need to have a more refined and user friendly means of classifying risk and vulnerability for MMPs. The research questions were formulated as follows:

1. What are the different patterns of mobility in the population groups living and/or working in or near forested areas?
2. What are the different risks and vulnerabilities linked to different work activities and mobility patterns?
3. How are exposure to malaria and access to health services affected by work activities and mobility patterns?
4. Can a useful classification system be developed to guide intervention strategies?

## Methods

The PMF was developed through an iterative process involving a review of the published and unpublished literature on MMPs in Cambodia, and the development and refinement of the framework based on the review and from expert opinion of researchers and programme managers in the Cambodian malaria programme. The framework was “tested” for face and content validity with national experts through a workshop approach. The process of the development of this framework (2012–2014) involved the following steps, supported through the NMCP:

1. Review of the published and grey literature on malaria and MMPs, and on interventions targeting those populations in Cambodia.
2. Analysis and synthesis of the information.
3. Presentation and discussion of the initial results at a consensus building workshops with experts to identify the major variables of risk, vulnerability.
4. Development of the framework components and indices.
5. Presentation of the framework to experts for face and content validity.

Firstly an extensive review of the relevant published and unpublished literature on malaria and migrant populations in Cambodia South-East Asia (neighbouring countries) was conducted. The aim was not to be exhaustive or systematic, but rather to ensure a comprehensive coverage of the literature relevant to the development of an evidence based framework and related indices for operationalization in the Cambodian context. The initial search of the published literature was carried out in PubMed for articles using the following keywords: migration or migrant or population movement and health or malaria. The search was further refined by focusing on those articles using the following keywords: either (1) risk or vulnerability or poverty or immunity, (2) exposure or transmission or housing or prevention or forest, (3) access or accessibility. The focus was on Cambodia and neighbouring countries, however when articles were not available for this geographic area, relevant articles covering other countries or regions were retained. The unpublished literature was identified through individual collections, searching web-sites and contacting individuals of relevant NGOs or research institutions working on MMP in Cambodia or in the GMS (including the World Health Organization, International Organization on Migration, Cambodian National Malaria Control Program, Malaria Consortium, London School of Hygiene and Tropical Medicine, Partners for Development, University Research Company, US Centre for Disease Control). The key information from the review of literature was synthesized in a report with a focus on previous frameworks related to MMPs; descriptions of risks and vulnerability; descriptions and evaluations of interventions.

Building on the literature and initial feedback from the consensus building workshop, the PMF for Cambodia was developed, comprising a categorization of the PMF itself, a description of MMP activity profiles and three indices which for operationalizing the PMF and activity profiles. The development of the PMF was based on the assumption that, the malaria ecosystem and transmission is closely related to forested areas in South East Asia [39]; therefore, the intensity, duration and frequency of the interaction/exposure with forest, itself influenced by mobility patterns and activities conducted by population in or near forested area, will condition the malaria incidence in the population.

The final outputs, which are described in the results section below, comprises of the PMF itself, MMP activity profiles and three indices for operationalizing the PMF and activity profiles. For each of the indices a simple scoring system was developed. As the nature of information available regarding MMP was mainly qualitative, ordinal scales (low–medium–high) and qualitative scoring (low = 1; medium = 2; high = 3) were used to define and quantify the indices. The source of information to attribute a value score to the variables for each population type or activity profile is based on a mix of experts opinion and field observations, quantitative results from the Cambodia Malaria Survey (CMS) 2010 [42] and qualitative studies undertaken in Cambodia recently [35].

## Results

The proposed PMF focuses on determinants affecting malaria epidemiology among MMP based on their mobility and migration status and on their main activities in relation to the forest (Table 1). As per previous frameworks, the PMF was developed along time and space dimensions. Along the temporal dimension, mobility pattern and migration status were categorized into three categories: local, mobile and migrant. The “local” population were defined as individuals residing in the area for more than 1 year and were included due to their circular mobility, usually into nearby forested areas for livelihood activities; the “mobile” population were defined as individuals residing in the area for <6 months; and migrants were defined as individuals residing in the area for more than 6 months and <1 year. Mobility was further broken down into “circulation” and “migration” movements. For “circulation”, the following criteria are considered: daily circulation (no overnight); periodic circulation (overnight to 1 week); seasonal circulation (1 week to 6 months). For “migration”: irregular migration (people residing for more than 6 months but moving out of the area) and long-term migration (new settlement over 6 months of stay). For the spatial dimension, population movement was only considered in relation to movement into forested areas, as the type of movement related to malaria risk. The forested areas were further characterized into type of forest i.e. foothills or upland forest. For distance travelled, the following criteria were used: short: <10 kms; medium: 10–100 kms; long: >100 kms and for the place of origin either, rural villages or urban area).

**Table 1:** Population movement framework

		Time				
Population type		Local population	Mobile population		Migrant population	
Movement type		Circulation (<6 months)			Migration (>6 months)	
Movement frequency		Daily (no overnight)	Periodic (up to 1 week)	Seasonal (1 week to 6 months)	Irregular	Long-term
Space						
*Short-range* Same commune-malaria area <10 kms	Rural village to foot hills	Farming, plantation	Farming- plantation	Farming, plantation		Farming, plantation New settlement Small business (shop)
	Rural village to upland forest	Farming, hunting, collecting, fishing, logging	Farming, hunting, collecting, fishing, logging	Farming, hunting, collecting, fishing, logging		Farming, plantation New settlement Small business (shop)
*Medium range* Same province usually non malaria area (10–100 kms)	Rural village to foot hills		Farming, plantation	Farming, plantation		Farming, plantation New settlement
	Rural village to upland forest		Hunting, logging, fishing Military, police	Hunting, logging, fishing Military, police Dam/road construction, mining	Hunting, logging, fishing Military, police Dam/road construction, mining	
*Long-range* Other province-often non malaria area >100 kms	Rural village to foot hills,		Visiting relatives, tourism	Farming, plantation,		Farming, plantation New settlement, trading
	Rural village or urban areas to upland forest		Farming-plantation, Hunting, logging, fishing Military, police	Hunting, logging, fishing Military, police, Dam/road construction, mining	Hunting, logging, fishing, Military, police, Dam/road construction, mining	Hunting, logging, fishing

The MMP activity profiles component of the framework (Table 2) was built upon an analysis of the range of activities conducted by individuals and groups working in and around forested areas. This includes agricultural activities, which can happen on a daily basis for local population and occur on short space range or can be seasonal and attract population from a longer space range, especially in plantation settings; construction activities, where a seasonal pattern can be observed (e.g. work halting in the wet season due to the difficulty in working or accessing remote area during the months of heavy rainfall); security activities including military forces which may travel over long distances from one area to another at various intervals of time depending on the political or military situation and the assignment of their unit; and hunting and fishing activities which can occur at almost all time periods and across all ranges of space. The latter includes local population going to the forest for hunting or fishing for their own livelihoods for short period of time, as well as people travelling hundreds of kilometres from other provinces to illegally hunt for rare animals or to collect precious woods, such as rosewood, for local or international markets.

**Table 2:** MMP activity profiles

Profile	Activities	Example
*Seasonal workers*	Agricultural activities occurring during planting season (end of dry season) and harvesting season (end of rainy season), usually in foothills/plains/valleys	Farming, rubber or cassava plantations
*Construction/mine workers*	Activities related to infrastructure construction or mining in forested areas, usually in upland forest/hills/valleys	Dam or road construction, gold or gem mines
*Forest workers*	Activities in heavily forested and remote areas in small mobile groups, usually in upland forest/hills	Gathering forest products, hunting, logging, fishing
*Security personnel*	Activities related to patrolling in forested border areas, including military, police, border patrol units	
*Visitors*	Tourism, visits to relatives which could include spending up to one week in or near the forest	Family event, national holiday, ecotourism

**Figure 1:**
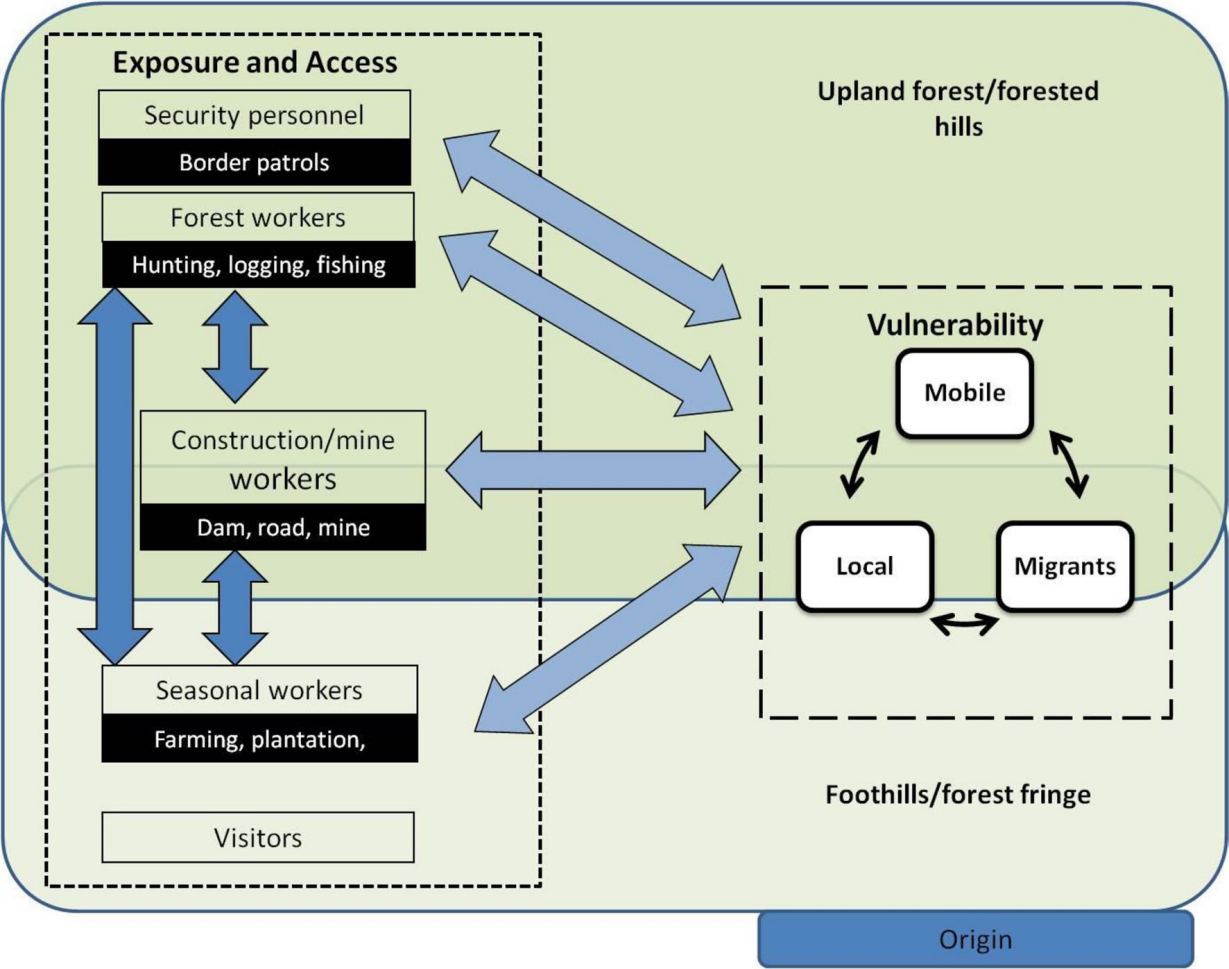
Visual representation of the PMF, including MMP types, activity profiles and indices. The largest frames describe the forest destination (the *upper* one the upland forest and forested hills, the *lower* frame the foothills and forest hills); on the *right* hand side, the long dash frame contains the vulnerability index affecting the types of MMP (local, mobile and migrants); on the *left* hand side, the* short dash* frame contains the exposure and access indices affecting the five activity profiles.* Arrows* illustrate the dynamic aspects of the PMF.

It is important to note that the resulting categories applied in the framework are not mutually exclusive and that individuals often fall into more than one group, for example seasonal workers who also go to work in the forest (i.e. they are also forest workers). This is further illustrated in Figure 1 showing the dynamic aspects and the interactions between the different activity profiles as well as between mobility patterns (local, mobile, migrant population) of individuals. As a visual representation of the PMF, this figure illustrates how mobility patterns will influence vulnerability of the population and how activity profiles will influence exposure to mosquito bites and access to health services. This figure illustrates movement of sub-groups of MMP between their origin (malaria or non-malaria area) and diverse eco-systems with various levels of malaria transmission. It shows how those different sub-groups might engage in forest related activities in different malaria risk areas and how people can potentially move between different profiles corresponding to different work activities. Although there is a need for definitions and categories in order to develop the strategy and implementation programme, it is important to keep in mind the dynamic and ever changing nature of movement of MMPs.

The PMF was operationalized by defining the following three indices which were used to group the key determinants of malaria risk in MMP. The three indices were: (1) vulnerability index, (2) exposure index and (3) access index, and then summated into the MMP malaria risk index.

**Figure 2:**
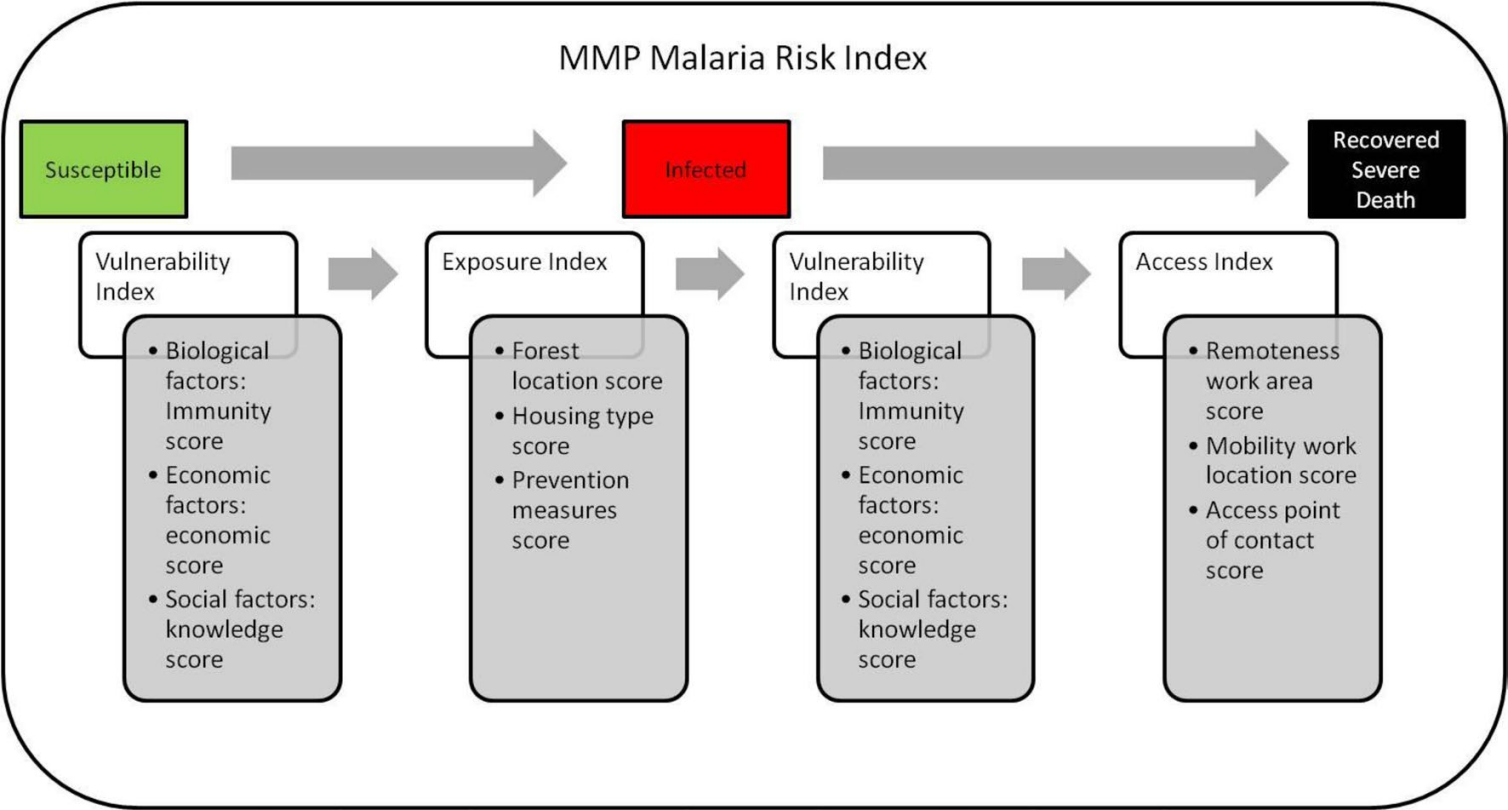
Diagram of the MMP malaria risk index. This diagram illustrates the phases from a susceptible patient to an infected one (morbidity), influenced by the vulnerability index and the exposure index and their respective underlying factors and from an infected patient to different outcomes (recovery, severity, mortality) influenced by the vulnerability index and the access index and their respective underlying factors.

Vulnerability, as considered here, is based on the variation in immunity, economic status and knowledge of malaria and of health services between mobile, migrant and local population and influences morbidity and mortality. Exposure, based on the variation of the location of work to the forest, housing types and the ownership and use of prevention measures influences the probability of being infected and therefore, morbidity. Access to health services and outreach of services to populations depends on the remoteness of the work area, the permanence of the work location and the availability of a point of contact and influences treatment-seeking behaviour and timely access to appropriate diagnosis and treatment, and therefore potential severity and mortality once infected. MMP malaria risk, in terms of morbidity and mortality, is therefore the results of the combined effects of these three main determinants (Figure 2). Each of these indices is described in more detail below.

### Vulnerability index: levels of vulnerability to malaria between different types of MMPs (mobile, migrant, local) (Table 3)

The vulnerability index is the summation of an immunity score, economic score and knowledge score. The vulnerability index aims to represent the various levels of vulnerability to malaria between different types of MMPs (mobile, migrant, local), assuming that the time spent in the new place at destination influences the level of vulnerability. The *immunity score*, is attributed based on the estimated immune status to malaria assuming that residence in a malaria area for less than a year results in no or low immunity. The *economic score* is attributed based on the estimated economic conditions assuming an association between migration, poverty and malaria. Recently arrived population in malaria areas have lower economic status than longer term residents and are therefore assigned a high economic score (corresponding to a higher vulnerability). The *knowledge score* is attributed based on the estimated knowledge of malaria transmission and prevention and of existing health services and assumes that recently arrived population in malaria areas, have lower knowledge of malaria and of health services than longer term residents and therefore the highest score (corresponding to a higher vulnerability). In summary, mobile population have a relatively higher vulnerability, than migrant population, who have a higher vulnerability than the local population.

**Table 3:** Vulnerability index

	Local population	Mobile population	Migrant population
Definition	Permanent resident for more than 1 year	Resident for <6 months	Permanent resident for more than 6 months and <1 year
Main residence	Village/house	Farm, plantation, company, outreach/mobile vendors/providers	Village/house
Biological factors			
Immune status	Low to medium	None or low	None or low
Immunity score	2	3	3
Economic factors			
Economic conditions	Low to medium	Low	Low to medium
Economic score	2	3	2
Social factors			
Knowledge malaria/health services	Medium to high	Low	Low to medium
Knowledge Score	1	3	2
Vulnerability index	5	9	7

### Exposure index: risk exposure to malaria based on the five activity profiles (Table 4)

In Cambodia, exposure to malaria is mainly dependent on the intensity, duration and frequency of interaction with the forest, and this in turn, is mainly conditioned by the type of activity in relation to the forest. Based on existing report and field experience in Cambodia, an exposure index was developed to express the risk of exposure to infected mosquito bites and summated three risks, namely: work location in relation to the forest; housing type; and the ownership and use of prevention measures. *The forest location score*, was developed whereby the closer the work location to the forest, the higher the forest location score. The *housing type score* was developed based on housing conditions. Forest workers have been observed to often sleep in the open in a hammock or in a basic tent (corresponding to a higher exposure), construction workers, seasonal workers and security personnel often live and sleep in huts, wooden barracks/houses or tents and visitors/long term residents usually stay in wooden or concrete houses (corresponding to a lower exposure). The *prevention measure score* was developed based on the likely levels of ownership and use of preventative measures according to MMP activity profile. As described in the background, forest workers often have very low ownership and use of LLINs (corresponding to higher exposure), construction workers, security personnel and seasonal workers often have nets provided by the company/employer or NGOs. In summary, forest workers have the highest exposure index, followed by construction/mine workers, security personnel and seasonal workers, while visitors have the lowest exposure index.

**Table 4:** Exposure index

	Forest workers (FW)	Construction workers (CW)	Security personnel (SP)	Seasonal workers (SW)	Visitors (V)
Main activities	Gathering forest products, fishing, hunting, logging	Dam or road construction, mining	Patrolling	Farming-chamkar, plantation	
Population type	Local, Mobile, Migrant	Mobile, Migrant	Mobile, Migrant	Local, Mobile, Migrant	Mobile
Work area	Upland forest, forested hills	Upland forest, forested hills	Border forest	Foot hills, plains, valleys	
Forest location Score	3	3	3	2	2
Housing type	Tents, none	Huts, barracks, tents	Huts, barracks, tents	Tents, huts	Wooden or concrete house
Housing type Score	3	2	2	2	1
Prevention measures use	Very low	Low	Low to medium	Low	Medium
Prevention measures Score	3	2	2	2	1
Exposure index	9	7	7	6	4

### Access index (Table 5)

Finally, an *Access index* was developed based on geographical accessibility, both in terms of the individual’s access to health service providers (demand) as well as the ability of health service providers to reach individuals (supply). A *remoteness work area score* was developed whereby forest workers, construction workers and security personnel who often work in remote border or cross-border areas are considered to have a higher remoteness score than seasonal workers who often work in forest fringes on plantations, and visitors/long term residents. A *mobility work location score* was developed to reflect the level of transience of the work place. Forest workers were considered to be highly mobile, and construction workers, security personnel and seasonal workers were attributed a medium score as they usually work in a fixed location but with some degree of mobility around it, or between multiple work locations,. The linked or unlinked character of workers to a company, a village or the government, allowing for a potential point of contact to deliver interventions, has been highlighted as an important variable affecting access [100]. *The access through linkage score*, was developed to reflect this. Forest workers were identified as usually not linked to any company or village, or because they are engaged in illegal activities they deliberately avoid contact. On the other hand construction workers, security personnel and seasonal workers would be linked to a company or a military base. In summary, forest workers have the highest access index (showing lower access), followed by construction/mine workers, security personnel, and seasonal workers, while visitors have the lowest access index (showing higher access).

**Table 5:** Access index

	Forest workers (FW)	Construction workers (CW)	Security personnel (SP)	Seasonal workers (SW)	Visitors (V)
Main activities	Hunting, fishing, logging, non-timber forest products	Dam or road construction, mining	Patrolling	Farming, plantation, chamkar	
Population type	Local, Mobile, Migrant	Mobile, Migrant	Mobile, Migrant	Local, Mobile, Migrant	Mobile
Work area	Upland forest, forested hills	Upland forest, forested hills	Border forest	Foot hills, plains, valleys	Variable
Remoteness work area	High	High to medium	High	Medium	Low
Remoteness work area score	3	3	3	2	1
Work location	Mobile	Fixed	Semi-mobile	Fixed	Fixed
Mobility work location	High	Medium	Medium–high	Medium	Low
Mobility work location score	3	2	2	2	1
Linkage	None or village for local population	Company	Government; military base	Farm owner/company	Village; guest houses/hotels
Access through linkage	Low	Low–medium	Medium	Medium–high	Medium–high
Access through linkage score	3	2	2	2	1
Access index	9	7	7	6	3

In order to develop the single MMP malaria risk index (Table 6) among different types of MMP (mobile; migrant; local) and across the different MMP activity profiles, the Vulnerability, Exposure and Access scores were arithmetically added. Based on this, the malaria risk score is the highest for mobile forest workers, followed by migrant forest workers, mobile construction workers; mobile security personnel and local forest workers. More generally, mobile population (except visitors) and forest workers rank the highest on the index, while local population (except forest workers) and visitors have the lowest rank.

**Table 6:** MMP Malaria risk index

			Vulnerability index					
			Mobile	Migrant	Local			
		Scores	9	7	5	Scores		
Exposure index	Forest workers	9	27	25	23	9	Forest workers	Access index
	Construction workers	7	23	21	19	7	Construction workers	
	Security personnel	7	23	21	19	7	Security personnel	
	Seasonal workers	6	20	18	16	5	Seasonal workers	
	Visitors	4	16	14	12	3	Visitors	

## Discussion

Broad definitions of migration and circulation (or mobility) have previously been described. These can be useful for characterizing HPM, better understanding population mobility on various temporal and spatial scales, and for defining vulnerability based on the diversity and disparity of MMP and related determinants. However, they are not always applicable for policy, planning or operational purposes. It is worth noting that the definitions of MMP used in this paper (local, mobile and migrant) represent a dynamic reality where overlap in time and interchange between categories is common. As shown in Figure 1, the triad local-mobile-migrant does not correspond to fixed attributes in time for each sub-group. Mobile population can travel to a new area for a few weeks and then return back to their place of origin for a few months, to then decide to go back and settle in this new area, becoming a migrant and then become part of the local population. Local residents may also be mobile for short period of time, qualifying them as mobile population. Similarly, seasonal workers after a few weeks of work in a cassava plantation can opportunistically work in a gold mine or on a dam construction while having some logging activities as forest workers.

As noted the development of the scoring system was pragmatic and influenced by the availability of data and expert opinion. Further iterations should include findings from ongoing research and programme activities. It is important to note that the criteria and cut-off points used in the definitions of mobility patterns are only indicative. In practice they vary and overlap depending on local situations. Mobility patterns and related forest activities can overlap or might happen sequentially for an individual or a group of persons over space and time. They are defined here, as such, to ensure clarity and may be adjusted when more information become available. Despite these limitations, the use of summary measures, as indices expressed in a score, although qualitative here, allows for ranking and classification of malaria risk among different types of MMP depending on their activities in different settings. Interventions should target the MMP with the highest malaria risk according to the PMF, namely mobile population and forest workers, although this population might be the most challenging to reach. Based on this, the PMF and indices can be populated with quantitative results from standardized field surveys (conducted among forest workers, seasonal workers, construction and mine workers and security personnel) to validate and refine the indices. Once this information is collected, ranking of malaria risk can be developed and the malaria risk index can be disaggregated into the sub-indices and their components to target and adjust malaria control interventions based on the needs identified in a given group and setting. For example, seasonal workers on a plantation might have good access to health services on site but high exposure due to poor housing or lack of prevention measures while forest workers might be using prevention measures, like impregnated hammock nets and repellents, but due to their remoteness, may not have access to health services. Specific interventions can then be provided based on the local situation. For example, in a given site with a high proportion of mobile population with high vulnerability, the health services might be orientated to strengthen programmes for health education about malaria to address the MMPs relatively poor knowledge and to increase outreach activities. The successful role of Mobile Malaria Workers in targeting MMP and the Village Malaria Workers program in Cambodia for the last decade could be also tailored to the most at risk MMP according to the PMF and risk index based on field data. Furthermore, mapping of the indices would allow for better geographical targeting of appropriate interventions by malaria control programme managers and implementers.

This framework was developed to inform the National Malaria Programme in their planning and development of a national strategy for malaria elimination among the MMPs in Cambodia [100]. It aims to develop an integrated approach to reach MMPs exposed to malaria, with prevention, diagnosis and treatment services, by involving non-health sector stakeholders from provincial to community level. Based on the PMF, intervention packages have been developed according to MMP types (covering their movement from their original residence to new places and their return back to origin) and to their activities. A special focus for these intervention packages is given at maximizing coverage, accessibility and acceptability of the interventions for each of the MMP groups, according to the local, geographical and epidemiological situation. Finally, although the PMF was developed in the context of Cambodia, the similarities of the situation regarding malaria and MMP in the GMS should allow for its application, with adaptation to the local context, in neighbouring countries.

## Conclusion

There is an urgent need to develop appropriate and accessible malaria services for MMP in different settings, in the context of the spread of artemisinin resistance and of malaria elimination in the GMS. In addition, in the context of global goals of universal coverage and access to basic health services, these remote and often marginalized populations (socially, economically or geographically) should be able to receive acceptable and affordable health care. Understanding the various nuanced categories of mobility amongst people and the situations which place them at risk of malaria is required, in order to effectively and efficiently meet their needs.

This framework has been used to develop more targeted behaviour change and outreach interventions for MMPs in Cambodia and its utility and effectiveness will be evaluated as part of those interventions. The PMF can assist in improved targeting of malaria (and other public health interventions) for Cambodia, and raises the need for other countries to also go beyond a simple labelling of risk groups to develop a better understanding of risk behaviours and vulnerabilities. However developing and maintaining this framework requires a broader set of data and skills than currently collected by routine health information systems in most countries, and demonstrates the need to integrate the analysis of data from a variety of sources including routine health information and research in order to meet the needs of programmes.

The implementation of the framework should be carefully evaluated to identify the changes in coverage, access, and effectiveness of the programme efforts to serve MMPs. The lessons learnt from this approach could assist improving the cost effectiveness and impact of the Cambodian programme and serve as a model for other countries to consider when planning programmes for MMPs.
